# Characteristics of Cognitive Behavioral Therapy for Older Adults Living in Residential Care: Protocol for a Systematic Review

**DOI:** 10.2196/resprot.9902

**Published:** 2018-07-04

**Authors:** Phoebe Chan, Sunil Bhar, Tanya E Davison, Colleen Doyle, Bob G Knight, Deborah Koder, Kenneth Laidlaw, Nancy A Pachana, Yvonne Wells, Viviana M Wuthrich

**Affiliations:** ^1^ Department of Psychological Sciences Swinburne University of Technology Melbourne Australia; ^2^ School of Health Sciences Swinburne University of Technology Melbourne Australia; ^3^ Faculty of Health Sciences Australian Catholic University Melbourne Australia; ^4^ School of Psychology and Counselling University of Southern Queensland Toowoomba Australia; ^5^ Royal Prince Alfred Hospital Sydney Australia; ^6^ Department of Clinical Psychology Norwich Medical School University of East Anglia Norwich United Kingdom; ^7^ School of Psychology The University of Queensland Brisbane Australia; ^8^ Lincoln Centre for Research on Ageing (Australian Institute for Primary Care and Ageing) La Trobe University Bundoora Australia; ^9^ Centre for Emotional Health Department of Psychology Macquarie University Sydney Australia

**Keywords:** cognitive behavioural therapy, older adults, residential care, delivery, characteristics, systematic review

## Abstract

**Background:**

The prevalence rates of depressive and anxiety disorders are high in residential aged care settings. Older adults in such settings might be prone to these disorders because of losses associated with transitioning to residential care, uncertainty about the future, as well as a decline in personal autonomy, health, and cognition. Cognitive behavioral therapy (CBT) is efficacious in treating late-life depression and anxiety. However, there remains a dearth of studies examining CBT in residential settings compared with community settings. Typically, older adults living in residential settings have higher care needs than those living in the community. To date, no systematic reviews have been conducted on the content and the delivery characteristics of CBT for older adults living in residential aged care settings.

**Objective:**

The objective of this paper is to describe the systematic review protocol on the characteristics of CBT for depression and/or anxiety for older adults living in residential aged care settings.

**Methods:**

This protocol was developed in compliance with the recommendations of the Preferred Reporting Items for Systematic Review and Meta-Analysis Protocols (PRISMA-P). Studies that fulfill the inclusion criteria will be identified by systematically searching relevant electronic databases, reference lists, and citation indexes. In addition, the PRISMA flowchart will be used to record the selection process. A pilot-tested data collection form will be used to extract and record data from the included studies. Two reviewers will be involved in screening the titles and abstracts of retrieved records, screening the full text of potentially relevant reports, and extracting data. Then, the delivery and content characteristics of different CBT programs of the included studies, where available, will be summarized in a table. Furthermore, the Downs and Black checklist will be used to assess the methodological quality of the included studies.

**Results:**

Systematic searches will commence in May 2018, and data extraction is expected to commence in July 2018. Data analyses and writing will happen in October 2018.

**Conclusions:**

In this section, the limitations of the systematic review will be outlined. Clinical implications for treating late-life depression and/or anxiety, and implications for residential care facilities will be discussed.

**Trial Registration:**

PROSPERO 42017080113; https://www.crd.york.ac.uk/PROSPERO/display_record.php?RecordID=80113 (Archived by WebCite at http://www.webcitation.org/70dV4Qf54)

**Registered Report Identifier:**

RR1-10.2196/9902

## Introduction

Older adults living in residential care settings have a high prevalence rate of depression and anxiety disorders. A systematic review of studies involving aged care residents from North America, Europe, Middle East, Australia, New Zealand, Africa, and Asia [1] reported that the prevalence of a major depressive disorder ranged from 4.8% (13/270) to 23.5% (12/51), whereas the prevalence of depressive symptoms ranged from 14% (99/708) to 81.8% (113/138). Another systematic review that examined the prevalence of anxiety in older adults living in residential aged care [2] found that the prevalence of anxiety disorders ranged from 3.2% (31/966) to 20% (20/100), whereas the prevalence of clinically significant anxiety symptoms ranged from 6.5% (3/46)to 58.4% (118/202). The most common anxiety disorders among aged care residents were generalized anxiety disorder and specific phobias.

The high prevalence of depression and anxiety in residential care settings may be attributed to several factors, including losses (eg, social connections and personal possessions) and changes (eg, lifestyle and health) involved in transitioning to living in a residential care facility [1,3,4]. Other factors associated with depression and anxiety in older adults living in residential care include multiple chronic health problems, chronic pain, functional impairment in basic activities of daily living (eg, bathing), functional impairment in instrumental activities (eg, managing finances), sensory impairments (eg, vision and hearing), cognitive decline, loneliness, negative life events, lack of social support, perceived inadequacy of care, perceived inability to master and control external environment, a low sense of purpose in life, and low perceived autonomy [5-7]. Another contributing factor could be the patterns of interactions between nursing staff and residents of care facilities. Baltes et al [8,9] reported that residents developed learned dependency when the social environment provided consistent and immediate support for dependent self-care behaviors (eg, residents who did not attempt to eat by themselves would likely receive immediate help from staff members, whereas those who attempted to eat independently would not receive praise, encouragement, or attention). Thus, learned dependency could have a negative impact on residents’ self-image and sense of control; subsequently, this could affect their psychological well-being.

To date, several approaches (eg, psychotherapy, pharmacological interventions, and music therapy) have been used to treat late-life depression and anxiety [10]. The efficacy of psychotherapeutic interventions, particularly cognitive behavioral therapies (CBT), has been demonstrated in several systematic reviews and meta-analyses conducted in the community and residential settings. CBT represents an approach that focuses on identifying and improving maladaptive behavioral and thinking patterns to assist clients in achieving goals. CBT includes a wide range of cognitive and behavioral techniques and is structured and goal-oriented.

Focusing on community-dwelling older adults, Wilson et al [11] reported that the efficacy of CBT in treating late-life depression was equivalent to or better than that of active control interventions (eg, visual imagery and education). Similarly, summarizing findings across community-based samples, Hendriks et al [12] reported that CBT was markedly more effective than waiting list and active control conditions (eg, usual care and supportive psychotherapy) in reducing anxiety symptoms in older adults diagnosed with anxiety disorders. In addition, a more recent meta-analysis [13] reported that CBT was markedly more effective in reducing anxiety symptoms in community-dwelling older adults compared with treatment as usual or being on a waiting list.

In a meta-analysis of the outcomes of psychotherapy for aged care residents, Cody and Drysdale [14] reported that psychotherapies were effective in reducing the symptoms of depression. In addition, they found that the effect of psychotherapies was comparable to that reported in pharmacotherapy trials with depressed older adults. Despite a lack of systematic reviews specifically examining CBT in residential care settings, studies such as those conducted by Anderson et al [15] and by Blair and Bird [16] found that CBT was effective and feasible for reducing depressive symptoms in older adults living in residential care.

Given the presence of multiple medical comorbidities and functional and cognitive decline, older adults in residential care might have different needs, clinical presentations, and perceptions of and responses to psychotherapies compared with community-dwelling older adults. In addition, the complexity of presentations might entail unique implementation models requiring interdisciplinary teamwork, sustainability of the intervention, flexibility of the environment, and support of the organization [17]. Specific programs, such as the Group, Individual, and Staff Therapy (GIST) [18] and the Behavioral Activities Intervention (BE-ACTIV) [19], have been developed for residential care settings. Nevertheless, no systematic review has been conducted on techniques and delivery characteristics of CBT when employed in residential care facilities. Such a systematic review is essential because it provides crucial information for the development of future CBT-based programs for residents in aged care. As highlighted by Kishita and Laidlaw [20] and Blair and Bird [16], identifying components of CBT programs that are specific to this population (eg, logistical issues such as how to approach residents, group size, timing, and duration of sessions) could help enhance treatment accessibility, acceptability, and outcomes. Moreover, by identifying the content of such protocols, that is, the strategies and techniques used to assist residents, clinicians may be better prepared to address the concerns of older adults living in residential aged care settings.

## Methods

### Objectives

This systematic review aims to describe the delivery and content characteristics of CBT for depression and anxiety for older adults living in residential aged care settings. This review adopts a broad definition of “older adults,” those aged ≥55 years.

### Protocol and Registration

This protocol was developed to comply with the recommendations of the Preferred Reporting Items for Systematic Review and Meta-Analysis Protocols (PRISMA-P) [21]. This systematic review will follow the guidelines of the Preferred Reporting Items for Systematic Reviews and Meta-Analyses (PRISMA) [22]. The review has been registered with the International Prospective Register of Systematic Reviews (PROSPERO, CRD 42017080113).

### Eligibility Criteria for the Review

#### Participants

In this review, those studies will be included, in which (1) participants were, at least, 55 years old, (2) were living in residential care (see definition below), and (3) standardized, valid measures (self-report questionnaire, observer rating, or clinical interview) were used to record the diagnosis and severity of depressive or anxiety disorders and symptom ratings of depression or anxiety. Samples of participants might include those living with dementia or mild cognitive impairment. Furthermore, studies that recruited participants aged <55 years but reported separate results for participants aged ≥55 years will be included.

#### Intervention

The target intervention is CBT for depressive symptoms, depressive disorders, anxiety symptoms, or anxiety disorders. Consistent with other studies [12,14], the types of psychotherapies considered to be CBT include behavioral therapy (including behavioral activation and exposure-based interventions), cognitive therapy, CBT, problem-solving therapy, rational emotive behavioral therapy, and mindfulness- and acceptance-based cognitive and behavioral therapies. Of note, studies that focused only on non-CBT interventions (eg, psychodynamic therapies, interpersonal therapies, and systemic therapies) or on psychological problems other than depression and anxiety will be excluded from this review. Moreover, studies will be excluded if their primary aim was to reduce disruptive behaviors associated with dementia or enhance memory.

#### Outcomes

The primary interest of this review is the delivery and content characteristics of CBT for residential aged care settings. Delivery characteristics refer to the (1) frequency, (2) duration, and (3) mode (group vs individual) of treatment, as well as to (4) whether others were involved in the delivery of treatment (eg, nurses, other facility staff members, and family members), and (5) whether treatment was delivered alone or in combination with other interventions. In contrast, the content characteristics refer to the therapeutic techniques used (eg, behavioral activation and cognitive restructuring). In addition, information on stakeholders’ reactions to these interventions will be described; such outcomes (whether assessed by a clinician, self-report, or an informant such as a staff member) will include participants’ satisfaction with the CBT intervention, staff members’ appraisal of the program, uptake rate, and attrition rate. Furthermore, these outcomes must be assessed with standardized, valid measures.

#### Setting

In this review, we will include studies conducted in residential care facilities and exclude those conducted only with community-dwelling older adults. Settings that are considered residential care facilities comprise nursing homes, aged care homes, residential aged care, and other communal living arrangements for older adults, where staff are employed to assist the residents with activities of daily living. Of note, studies conducted in retirement villages or retirement homes or hostels, where staff are only employed as on-site managers but do not provide care will be excluded.

#### Types of Studies

We will include empirical, quantitative studies that fulfill the criteria mentioned above. These studies could be randomized or quasi-randomized controlled trials, clinical controlled trials, cluster-randomized trials, cross-over trials, or case studies. However, commentaries and theoretical papers that describe protocols that have not been applied in residential aged care settings will be excluded.

#### Report Characteristics

We will include full-text papers written in English with no restrictions on the geographical location or year of publication.

### Search Methods for Identifying Studies

We will be conducting a systematic search of the following databases to identify published studies: the Cochrane Library (including the Cochrane Central Register of Controlled Trials (CENTRAL) and the specialized registers of the Common Mental Disorders Group and the Dementia and Cognitive Improvement Group), Medical Literature Analysis and Retrieval System Online (MEDLINE), EMBASE, PubMed, PsycINFO, Cumulative Index of Nursing and Allied Health Literature (CINAHL), Abstracts in Social Gerontology (EBSCO), AgeLine (EBSCO), Social Services Abstracts (ProQuest), Sociological Abstracts (ProQuest), the World Health Organization’s trials portal (ICTRP), and ClinicalTrials.gov. Next, unpublished studies will be identified by searching the ProQuest Dissertations and Theses database, Open Access Theses and Dissertations, and Open Grey. In addition, reference lists of all included studies will be examined and a citation search on the Web of Science will be conducted to identify relevant studies that might have been missed in the database searches. Furthermore, we will correspond with the authors of these studies, if it is feasible to do so, when more information on particular studies is required.

The search terms are arrived at by adapting those used by Cody and Drysdale [14] and Hendriks et al [12]. A sample search strategy for database searching will be as follows:


(“depress*” OR “dysthym*” OR “adjustment” OR “mood” OR “affective” OR “anxiety” OR “anxious” OR “worry” OR “phobi*” OR “panic” OR “obsess*” OR “compulsi*” OR “posttraumatic” OR “PTSD” OR “OCD”) AND

(“cognitive therapy*” OR “behavi* therapy*” OR “cognitive behavi* therapy*” OR “mindfulness-based therapy*” OR “acceptance commitment therapy*” OR “acceptance-based therapy*” OR “relaxation training” OR “activity scheduling” OR “cognitive restructuring”) AND

(“long-term care” OR “residential aged care” OR “aged care” OR “nursing home*” OR “assisted living” OR “care facility*” OR “residential home*” OR “care home*” OR “residential care”)

### Data Collection and Analysis

#### Selection of Studies

In this review, two reviewers will be involved in the process of selecting studies to ensure that the judgments are reproducible [23]. They will independently examine titles and abstracts of the records retrieved from the database search to remove obvious irrelevant reports. Then, the reviewers will screen the full text of the potentially relevant studies to assess their eligibility for inclusion. While one reviewer will screen all the records and papers, the second reviewer will screen, at least, 25% of them. Discrepancies between the reviewers will be resolved by discussion and consensus, and if necessary, by arbitration of a third reviewer. All initial levels of the agreement will be reported. In addition, the PRISMA flow diagram will be used to record the process of selection, as well as the numbers of records, full-text papers, and studies resulted from each stage.

#### Data Extraction and Management

We will use a pilot-tested data collection form to extract and record data from the included studies. The data extracted will comprise the following: publication information (eg, authors, title, journal, publication type, and geographical location wherein the study was conducted); study design (eg, randomized or quasi-randomized controlled trial, clinical controlled trial, cluster-randomized trial, cross-over trial, or case studies); participants’ characteristics (eg, sample size, age, gender, disorders or symptoms of depression and anxiety, cognitive abilities in terms of Mini Mental State Examination (MMSE) or other cognitive screen scores, and diagnoses or symptoms of dementia); details of the delivery characteristics of intervention (eg, frequency and duration of treatment, individual or group format, involvement of staff, families, or friends in delivering treatments, additional treatment models); details of the content characteristics of intervention (eg, behavioral activation and cognitive restructuring); and stakeholders’ reactions reported in the studies (eg, participants’ satisfaction with the intervention, uptake rate, attrition rate, and staff appraisal of the program).

Two independent reviewers will extract data from all the selected studies. Discrepancies between reviewers will be resolved by discussion and consensus, and if necessary, by arbitration of a third reviewer. Furthermore, if additional information is required, we will contact the study authors.

#### Assessment of Methodological Quality of Included Studies

We will use the Downs and Black [24] instrument, which can be used for randomized and nonrandomized controlled trials, to assess the methodological quality of the included studies. The checklist comprises the following five domains: reporting biases, external validity, biases in the measurement of the intervention and the outcome, biases in selecting participants, and statistical power. In this review, we will report the overall score and the score for each of the domains. Two reviewers will assess all the selected studies. Discrepancies between the reviewers will be resolved by discussion and consensus, and if necessary, by arbitration of a third reviewer.

#### Data Synthesis

In a table format, we will summarize and present the delivery and the content characteristics of different CBT programs of the included studies. While delivery characteristics refer to how the interventions were approached in residential care settings, the content characteristics refer to the strategies that therapists used with or taught the residents (as detailed above). Furthermore, we will highlight shared components between different programs and components that have been designed particularly for individual groups of residents.

## Results

Systematic searches are expected to commence in May 2018. Data extraction is expected to commence in July 2018. Data analyses and writing will happen in October 2018.

## Discussion

We will outline the limitations of this systematic review. For example, the studies reviewed might be of poor quality or insufficiently reported to allow for a full audit of relevant variables. Moreover, similar treatments might be labeled inconsistently across studies. We will discuss the clinical implications for treating depression and anxiety in older adults living in residential care settings. Furthermore, we will consider the delivery and content of CBT within such settings to facilitate further development of such treatments across the sector.

## Abbreviations

CBT: cognitive behavioral therapy
CENTRAL: Cochrane Central Register of Controlled Trials
CINAHL: Cumulative Index of Nursing and Allied Health Literature
EBSCO: Elton B Stephens CO (company)
ICTRP: International Clinical Trials Registry Platform
MMSE: Mini Mental State Examination
PRISMA: Preferred Reporting Items for Systematic Review and Meta-Analysis
